# Cooperation of endogenous and exogenous reactive oxygen species induced by zinc peroxide nanoparticles to enhance oxidative stress-based cancer therapy

**DOI:** 10.7150/thno.39831

**Published:** 2019-09-23

**Authors:** Li-Sen Lin, Jun-Feng Wang, Jibin Song, Yijing Liu, Guizhi Zhu, Yunlu Dai, Zheyu Shen, Rui Tian, Justin Song, Zhantong Wang, Wei Tang, Guocan Yu, Zijian Zhou, Zhen Yang, Tao Huang, Gang Niu, Huang-Hao Yang, Zhi-Yi Chen, Xiaoyuan Chen

**Affiliations:** 1 Department of Ultrasound Medicine, Laboratory of Ultrasound Molecular Imaging, the Third Affiliated Hospital of Guangzhou Medical University, Guangzhou 510150, China; 2 Laboratory of Molecular Imaging and Nanomedicine (LOMIN), National Institute of Biomedical Imaging and Bioengineering (NIBIB), National Institutes of Health (NIH), Bethesda, Maryland 20892, United States; 3 Department of Ultrasound, the First Affiliated Hospital of Harbin Medical University, Harbin, Heilongjiang 150076, China; 4 MOE Key Laboratory for Analytical Science of Food Safety and Biology, College of Chemistry, Fuzhou University, Fuzhou 350108, China; 5 Department of Radiology, the Fourth Hospital of Harbin Medical University, Harbin, Heilongjiang 150076, China

**Keywords:** reactive oxygen species, zinc peroxide nanoparticles, pH-responsiveness, magnetic resonance imaging, cancer therapy

## Abstract

Reactive oxygen species (ROS)-generating anticancer agents can act through two different mechanisms: (i) elevation of endogenous ROS production in mitochondria, or (ii) formation/delivery of exogenous ROS within cells. However, there is a lack of research on the development of ROS-generating nanosystems that combine endogenous and exogenous ROS to enhance oxidative stress-mediated cancer cell death.

**Methods:** A ROS-generating agent based on polymer-modified zinc peroxide nanoparticles (ZnO_2_ NPs) was presented, which simultaneously delivered exogenous H_2_O_2_ and Zn^2+^ capable of amplifying endogenous ROS production for synergistic cancer therapy.

**Results:** After internalization into tumor cells, ZnO_2_ NPs underwent decomposition in response to mild acidic pH, resulting in controlled release of H_2_O_2_ and Zn^2+^. Intriguingly, Zn^2+^ could increase the production of mitochondrial O_2_·^-^ and H_2_O_2_ by inhibiting the electron transport chain, and thus exerted anticancer effect in a synergistic manner with the exogenously released H_2_O_2_ to promote cancer cell killing. Furthermore, ZnO_2_ NPs were doped with manganese *via* cation exchange, making them an activatable magnetic resonance imaging contrast agent.

**Conclusion:** This study establishes a ZnO_2_-based theranostic nanoplatform which achieves enhanced oxidative damage to cancer cells by a two-pronged approach of combining endogenous and exogenous ROS.

## Introduction

Reactive oxygen species (ROS) including hydrogen peroxide (H_2_O_2_), superoxide anion radical (O_2_·^-^), singlet oxygen, and hydroxyl radical can damage lipids, proteins, and DNA, which would lead to cell death when the ROS level exceeds the cellular antioxidant capacity, a condition referred to as oxidative stress 1-6. Cells constantly generate endogenous ROS as by-products of aerobic metabolism in mitochondria, and maintain redox homeostasis by controlling the balance between ROS formation and scavenging 7. More importantly, it has been found that cancer cells show elevated mitochondrial ROS production due to oncogenic stimulation and active metabolism 8-10. Even though cancer cells enhance antioxidant capacity to counterbalance the overproduction of ROS, their ROS levels are still closer to the toxicity threshold than that of normal cells, making them more susceptible to further ROS formation induced by exogenous agents 11,12. Therefore, the development of ROS-generating agents that can effectively raise intracellular ROS levels above the toxicity threshold and cause oxidative stress-mediated cell death would be of great interest in cancer therapy.

There are two possible working mechanisms for ROS-generating anticancer agents: (i) enhancement of endogenous ROS production, and (ii) formation/delivery of exogenous ROS. On the one hand, it is well known that mitochondria are the main source of endogenous ROS, and that inhibition of mitochondrial electron transport chain (ETC) can promote the production of O_2_·^-^ and H_2_O_2_ by increasing leakage of electrons to oxygen (O_2_) at complexes I and III of the ETC 13,14. Therefore, several ETC inhibitors have been used to kill tumor cells through endogenous ROS-mediated apoptosis 15. On the other hand, anticancer agents with exogenous ROS-generating ability, such as photodynamic and sonodynamic sensitizers, are attracting more and more attention 16-21. In particular, stimuli-responsive nanoplatforms have been actively explored for the formation of exogenous ROS in tumor cells 22-28. However, to the best of our knowledge, there is a paucity of studies regarding the design of ROS-generating nanosystems that combine endogenous and exogenous ROS to potentiate oxidative stress-induced cancer cell death.

**Figure 1:**
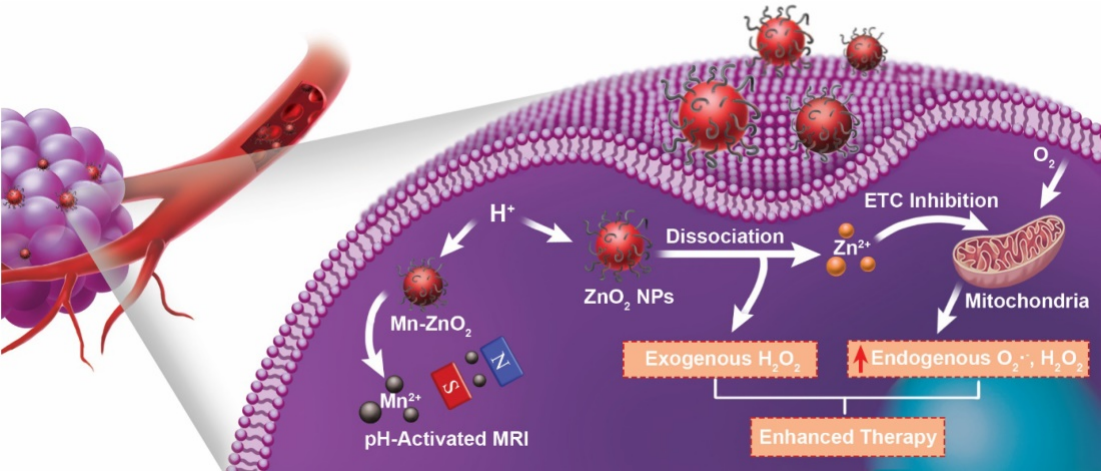
Schematic illustration of theranostic ZnO_2_ NPs for MRI and enhanced oxidative stress-based cancer therapy. Upon endocytosis by tumor cells, ZnO_2_ NPs undergo dissociation in response to mild acidic pH, causing the release of H_2_O_2_ and Zn^2+^. The exogenously released H_2_O_2_ and Zn^2+^, that can increase the production of mitochondrial O_2_·^-^ and H_2_O_2_ by inhibiting the electron transport chain (ETC), act synergistically to promote cancer cell killing through the cooperation of endogenous and exogenous ROS. Moreover, Mn-doping *via* partial cation exchange imparts ZnO_2_ NPs with pH-activated MRI contrast ability.

Herein, we report a novel ROS-generating agent based on zinc peroxide nanoparticles (ZnO_2_ NPs), which simultaneously delivers exogenous H_2_O_2_ and Zn^2+^ with the capability of intensifying endogenous ROS production for synergistic cancer therapy (Figure 1). The ZnO_2_ NPs were stable at neutral pH but decomposed to Zn^2+^ and H_2_O_2_ at mild acidic pH. In fact, it has been proved that zinc exerts its antitumor effects through inhibition of ETC and consequent elevation of mitochondrial ROS production 29-34. As compared to conventional zinc ionophores that have been utilized to increase Zn^2+^ accumulation in cancer cells 35-37, controlled Zn^2+^ delivery systems possess the potential for providing desirable therapeutic outcomes with reduced side effects. In addition, although formation of exogenous H_2_O_2_ within cells has been recognized as a promising strategy for tumor therapy 38,39, there has been little work on the preparation of H_2_O_2_-releasing systems with stimuli-responsive property. With these considerations in mind, we designed and synthesized poly(vinylpyrrolidone) (PVP)-modified ZnO_2_ NPs as a pH-triggered ROS-generating agent. Upon internalization in tumor cells, ZnO_2_ NPs would release exogenous H_2_O_2_ and Zn^2+^ able to promote endogenous ROS production in mitochondria, which functioned in a synergistic manner to enhance oxidative stress-based cancer cell killing. The vulnerability of tumor cells to additional ROS and the pH-responsiveness of ZnO_2_ NPs enabled this ZnO_2_-based ROS-generating anticancer agent to achieve good therapeutic efficacy with low side effects. Moreover, the ZnO_2_ NPs was successfully doped with paramagnetic manganese (Mn) *via* a cation-exchange method. The pH-stimulated Mn^2+^ release during the decomposition of Mn-doped ZnO_2_ (Mn-ZnO_2_) NPs endowed them with activatable magnetic resonance imaging (MRI) contrast ability, which is useful to monitor the dissociation of ZnO_2_ NPs as well as the subsequent therapeutic process. This study not only highlights the great potential of ZnO_2_ nanomaterials in cancer theranostics, but also provides a paradigm for designing stimuli-responsive ROS-generating nanoagents that exacerbate oxidative damage to cancer cells through collaboration between endogenous and exogenous ROS.

## Materials and methods

### Materials

Zinc acetate (Zn(OAc)_2_, 99.99%), polyvinylpyrrolidone (PVP, Mw = 10000), hydrogen peroxide (H_2_O_2_, 30 wt. % in H_2_O), manganese(II) chloride (MnCl_2_, 99%), zinc chloride (ZnCl_2_, 99.999%), zinc oxide nanoparticles (ZnO NPs, 20 wt. % in H_2_O), zinquin ethyl ester (95%), 3-(*N*-morpholino)propanesulfonic acid (MOPS, 99.5%), sodium acetate (99%), acetic acid (99.7%), 2',7'-dichlorofluorescin diacetate (DCFH-DA, 97%), thiazolyl blue tetrazolium bromide (MTT, 97.5%), and propidium iodide (PI, 94%) were purchased from Sigma-Aldrich. Apoptosis kit with annexin V-FITC and PI, hydrogen peroxide assay kit, and calcein-AM were obtained from Fisher Scientific.

### Synthesis of PVP-modified ZnO_2_ NPs

0.1 g of Zn(OAc)_2_ and 0.1 g of PVP were dissolved in 5.0 mL of water. Then, 0.5 mL of H_2_O_2_ was added quickly with vigorous stirring. After reaction for 24 h, the resulting PVP-modified ZnO_2_ NPs were washed several times and then re-dispersed in water.

### Synthesis of Mn-ZnO_2_ NPs

The Mn-doping was achieved by a cation-exchange approach. Briefly, the aqueous solution of ZnO_2_ NPs was mixed with the same volume of MnCl_2_ with different Mn concentrations. After stirring at room temperature for 4 h, the obtained Mn-ZnO_2_ NPs were collected by centrifugation (15000 rpm, 15 min).

### Decomposition of ZnO_2_ NPs

The release of Zn^2+^ and H_2_O_2_ from ZnO_2_ NPs was determined by dialysis. The aqueous solution of ZnO_2_ NPs was dialyzed against acetate buffer (pH 5.5) or MOPS buffer (pH 7.4). The Zn^2+^ was measured by inductively coupled plasma optical emission spectrometry (ICP-OES) and the H_2_O_2_ was measured by using a hydrogen peroxide assay kit.

### Intracellular release of Zn^2+^

Zinquin ethyl ester, a cell-permeable fluorescent probe for Zn^2+^, was used to evaluate intracellular Zn^2+^ release. After incubation with ZnO_2_ NPs for 4 h, U87MG cells were stained with 25 μM zinquin ethyl ester at 37 °C for 30 min. Then, the fluorescence images were collected by an Olympus IX81 fluorescence microscope.

### Oxidative stress assessment

Intracellular oxidative stress was determined by using DCFH-DA as a probe. U87MG cells seeded in 6-well plates (2×10^5^ cells/well) were exposed to H_2_O_2_, ZnCl_2_, H_2_O_2_ plus ZnCl_2_, or ZnO_2_ NPs for 4 h. After staining with 10 μM DCFH-DA at 37 °C for 30 min, the fluorescence images were obtained.

### *In vitro* cancer therapy

To demonstrate the synergistic anticancer effect of Zn^2+^ and H_2_O_2_, U87MG cells (5×10^3^ cells/well) seeded in 96-well plates were incubated with H_2_O_2_, ZnCl_2_, or H_2_O_2_ plus ZnCl_2_ (molar ratio, 1:1) for 24 h. Then, the cell viability was measured by MTT assay. Similarly, the *in vitro* anticancer activity of PVP-modified ZnO_2_ NPs was examined.

### Magnetic resonance imaging

To confirm the pH-activated MRI contrast effect, the Mn-ZnO_2_ NPs were dispersed in different buffer solutions for 4 h, and then the samples were imaged by an MRI scanner. For *in vivo* MRI, U87MG tumor-bearing mice were injected with Mn-ZnO_2_ NPs (200 μL, [Mn] = 1 mM) through the tail vein. Then, the *T*_1_-weighted MRI images were obtained by an MRI scanner.

### *In vivo* tumor growth inhibition

Mice bearing U87MG tumors (~50 mm^3^) were injected intravenously with different formulations every other day for four doses, including (1) saline, (2) H_2_O_2_, (3) ZnCl_2_, and (4) ZnO_2_ NPs. Each dose of H_2_O_2_: 0.05 mmol/kg, ZnCl_2_: 0.05 mmol/kg, ZnO_2_ NPs: 5 mg/kg. The tumor volume and body weight of each mouse were recorded every 2 days.

## Results and discussion

**Figure 2:**
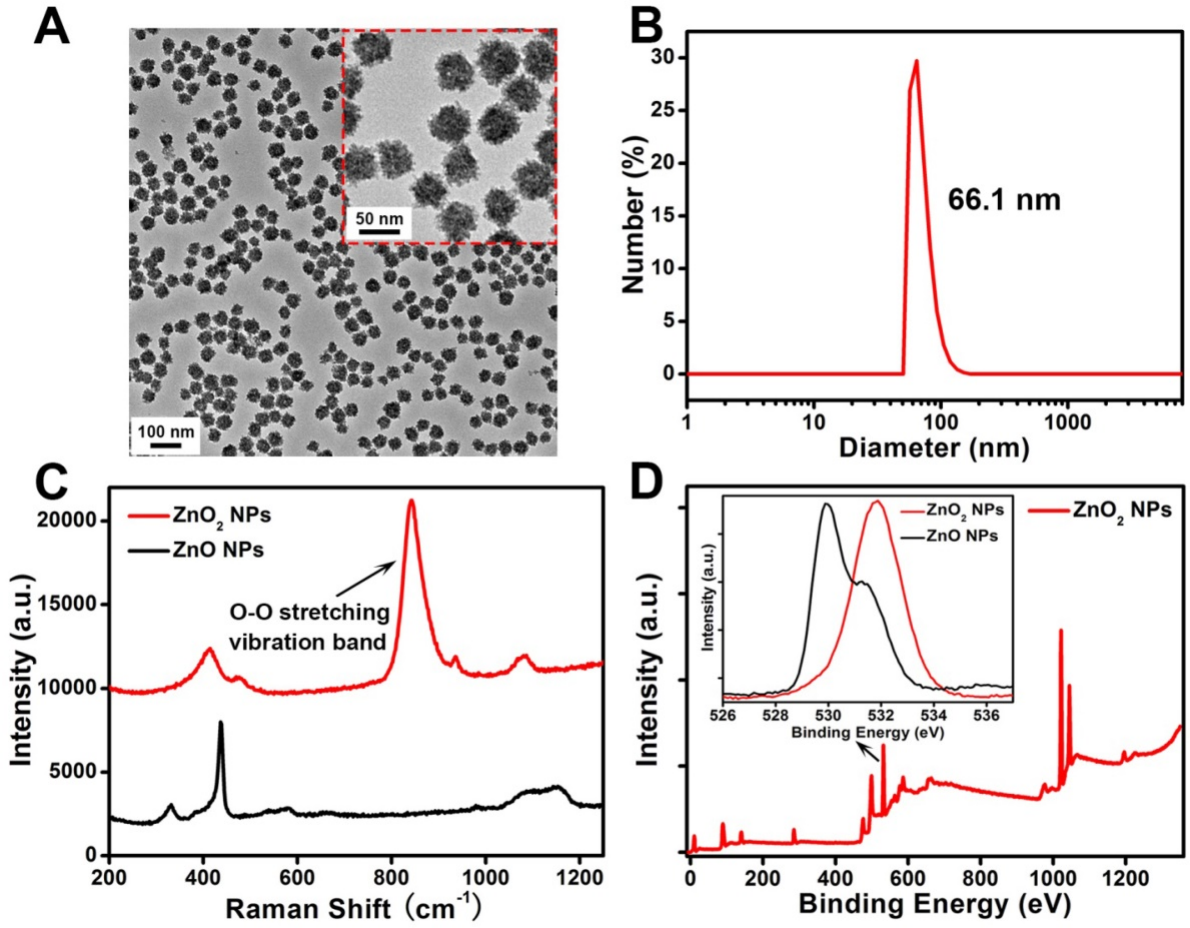
(A) TEM image and (B) DLS data of ZnO_2_ NPs. (C) Raman spectra of ZnO NPs and ZnO_2_ NPs. (D) XPS survey spectra and O 1s peak (inset) of ZnO_2_ NPs.

The ZnO_2_ NPs were fabricated by reacting zinc acetate with H_2_O_2_ in the presence of PVP at room temperature for 24 h. As shown in the transmission electron microscopy (TEM) image (Figure 2A), the as-prepared ZnO_2_ NPs had a uniform particle size of about 50 nm. Dynamic light scattering (DLS) results revealed that the average hydrodynamic diameter of ZnO_2_ NPs was ~66.1 nm (Figure 2B). In contrast to zinc oxide nanoparticles (ZnO NPs), ZnO_2_ NPs exhibited a strong band at 840 cm^-1^ in the Raman spectra assigned to O-O stretching vibration (Figure 2C) 40, clearly demonstrating the existence of peroxide ions (O_2_^2-^). Moreover, the successful synthesis of ZnO_2_ NPs was further confirmed by X-ray photoelectron spectroscopy (XPS). An O 1s peak with binding energy of 532 eV corresponding to O_2_^2-^ was observed for the ZnO_2_ NPs, whereas the O 1s peak of ZnO NPs was located at 530 eV attributed to O^2-^ (Figure 2D, Figure S1) 41.

**Figure 3:**
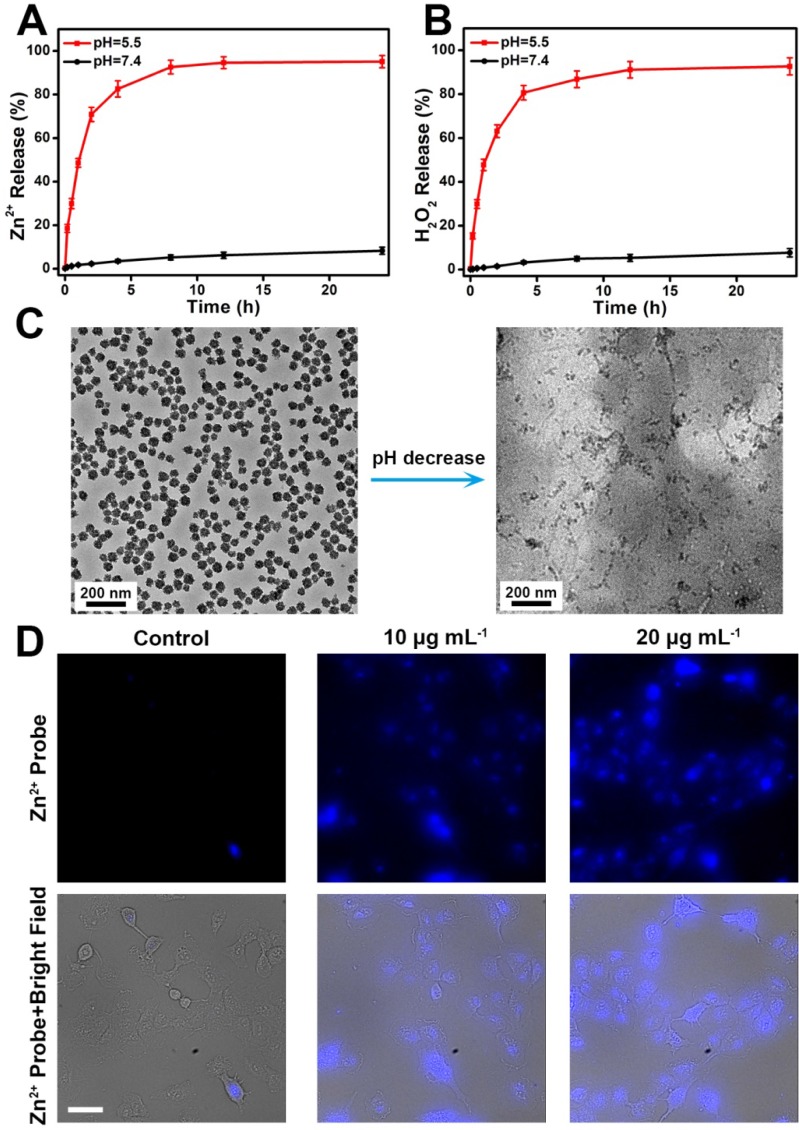
Release profiles of (A) Zn^2+^ and (B) H_2_O_2_ from ZnO_2_ NPs at different pH values. (C) TEM images of ZnO_2_ NPs after 2 h of incubation in pH 7.4 (left) and pH 5.5 (right) buffer solutions. (D) Fluorescence images of zinquin ethyl ester-stained U87MG cells after incubation with different concentration of ZnO_2_ NPs for 4 h. Scale bar, 50 μm.

To demonstrate the pH-dependent dissociation behavior of ZnO_2_ NPs, we measured the release of Zn^2+^ and H_2_O_2_ from ZnO_2_ NPs under neutral (pH 7.4) or acidic (pH 5.5) conditions. It can be seen in Figure 3A,B that both Zn^2+^ and H_2_O_2_ were gradually released from the ZnO_2_ NPs at pH 5.5. In contrast, the release rates were relatively slow in pH 7.4 buffer solution. As shown by TEM (Figure 3C), ZnO_2_ NPs were stable at pH 7.4 but degraded at pH 5.5. Thus, the accelerated release in mild acidic environment can be ascribed to the pH-responsive degradation of ZnO_2_ NPs, which is favorable for *in vivo* tumor treatment since the acidic endo/lysosomes (pH 5.0-6.0) of cancer cells can trigger the release of H_2_O_2_ and Zn^2+^ on demand. Then, the decomposition of ZnO_2_ NPs in cancer cells was confirmed by evaluating the intracellular release of Zn^2+^ with a cell-permeable fluorescent Zn^2+^ probe, zinquin ethyl ester 42-45. As expected, ZnO_2_ NPs-incubated U87MG cancer cells exhibited stronger blue fluorescence than control untreated cells, and the blue fluorescence increased with increasing concentration of ZnO_2_ NPs (Figure 3D, Figure S2), demonstrating the intracellular degradation of ZnO_2_ NPs.

**Figure 4:**
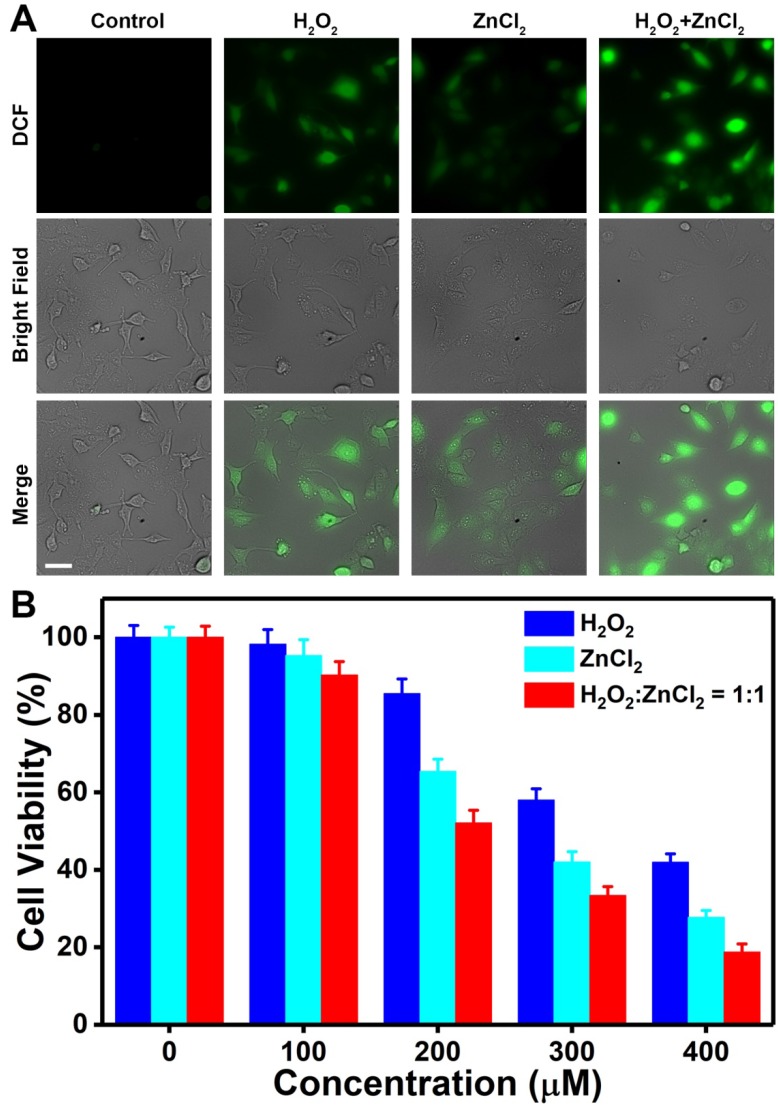
(A) DCF fluorescence of U87MG cells after different treatments. [H_2_O_2_] = 200 μM, [ZnCl_2_] = 200 μM. Scale bar, 50 μm. (B) Cell viability after 24 h of exposure to H_2_O_2_, ZnCl_2_, or H_2_O_2_ plus ZnCl_2_ (molar ratio, 1:1).

To determine whether Zn^2+^ and H_2_O_2_ could exert anticancer activity in a synergistic manner, we assessed their ability to induce oxidative stress and cytotoxicity. First, the intracellular oxidative stress caused by H_2_O_2_ or Zn^2+^ was examined by employing 2',7'-dichlorofluorescin diacetate (DCFH-DA) as the probe. After being deacetylated by cellular esterases, DCFH-DA is converted to DCFH that can interact with ROS to generate fluorescent 2',7'-dichlorofluorescein (DCF) 46-51. As shown in Figure 4A, U87MG cells incubated with either H_2_O_2_ or ZnCl_2_ displayed significantly greater DCF fluorescence compared with untreated control cells, implying the occurrence of oxidative stress in cancer cells upon H_2_O_2_ or Zn^2+^ exposure. The Zn^2+^-dependent oxidative stress could be mainly attributed to the increased mitochondrial ROS production 29-34. Moreover, the DCF fluorescence in U87MG cells co-incubated with H_2_O_2_ and ZnCl_2_ was higher than that in cells incubated with H_2_O_2_ or ZnCl_2_ alone, which indicated the combined effect of H_2_O_2_ and Zn^2+^ on amplification of oxidative stress. The oxidative stress-mediated cytotoxicity of H_2_O_2_ and Zn^2+^ was further evaluated by MTT assay. As expected, both H_2_O_2_ and ZnCl_2_ exhibited concentration-dependent toxicity (Figure 4B). More importantly, the viability of U87MG cells co-treated with H_2_O_2_ and ZnCl_2_ at a molar ratio of 1:1 was lower than that of cells treated with ZnCl_2_ or H_2_O_2_ alone, demonstrating the synergistic anticancer effect of H_2_O_2_ and Zn^2+^. It has been reported that mitochondria, one of the most important organelles responsible for energy metabolism, are particularly vulnerable to oxidative damage as they are constantly exposed to high levels of endogenous ROS 52. Therefore, the synergy between H_2_O_2_ and Zn^2+^ is probably because large amount of exogenously released H_2_O_2_ is able to disturb cellular redox homeostasis and then the enhanced mitochondrial ROS production resulting from inhibition of ETC by Zn^2+^ can lead to the impairment of mitochondria. These results suggested that exogenous H_2_O_2_ and Zn^2+^ with the capability of promoting endogenous ROS production could function cooperatively to enhance oxidative stress-induced tumor cell death.

**Figure 5:**
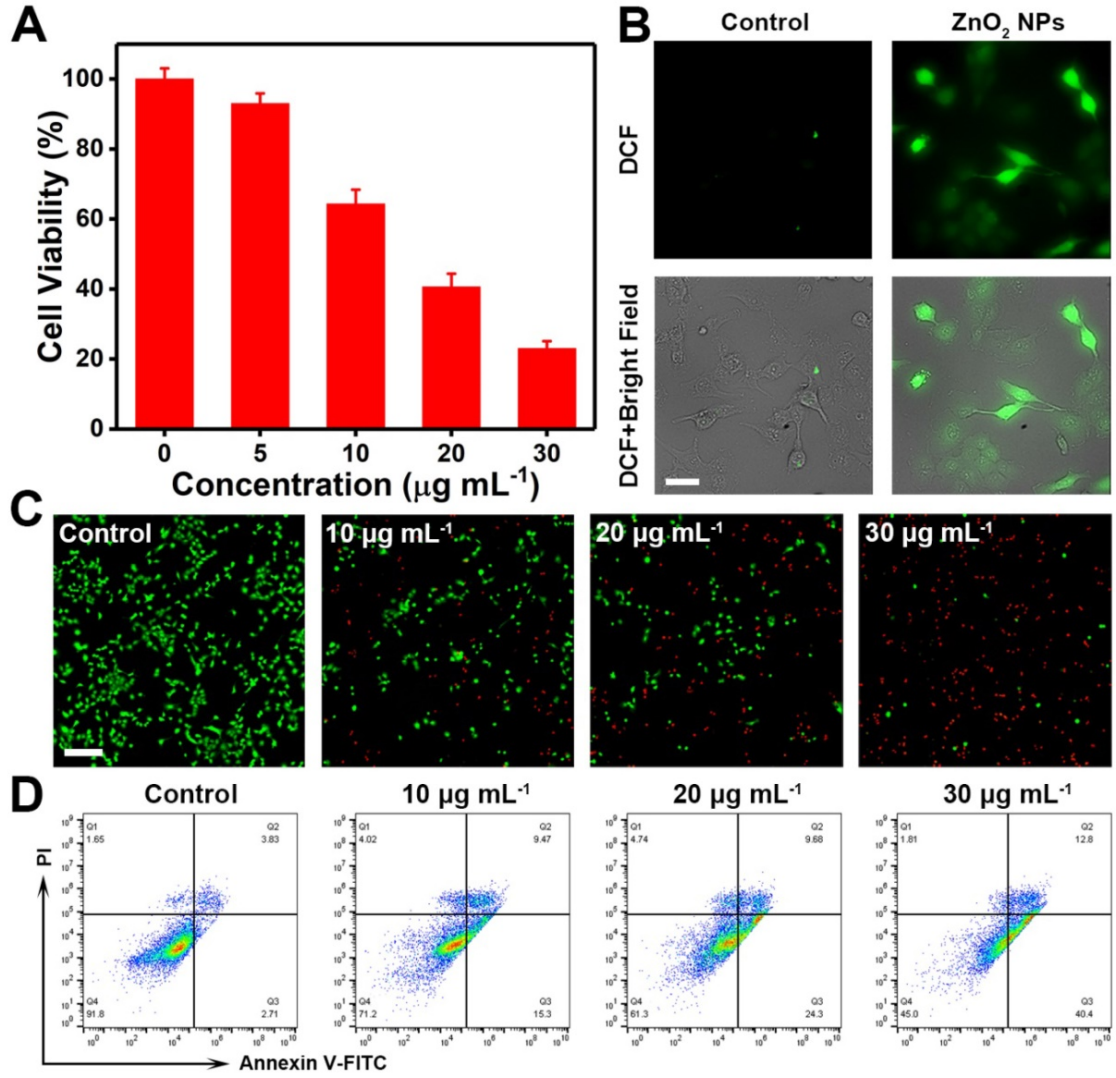
(A) *In vitro* anticancer activity of ZnO_2_ NPs after 24 h of incubation. (B) DCF fluorescence of U87MG cells after 4 h of incubation with 10 μg mL^-1^ ZnO_2_ NPs. Scale bar, 50 μm. (C) Calcein-AM (green, live cells) and PI (red, dead cells) co-stained fluorescence images of cells treated with different concentrations of ZnO_2_ NPs for 24 h. Scale bar, 100 μm. (D) Flow cytometry data showing apoptosis in U87MG cells after exposure to ZnO_2_ NPs for 12 h.

Next, the *in vitro* anticancer effect of PVP-modified ZnO_2_ NPs was examined. As can be seen in Figure 5A, the PVP-modified ZnO_2_ NPs exerted remarkable therapeutic efficacy against cancer cells, which could be ascribed to the pH-responsive release of H_2_O_2_ and Zn^2+^ from ZnO_2_ NPs in acidic endo/lysosomes and the subsequent occurrence of oxidative stress (Figure 5B). In addition, ZnO_2_ NPs exhibited a time- and dose-dependent cytotoxicity (Figure S3). To further confirm ZnO_2_ NPs-caused cancer cell death, the fluorescent indicators calcein-AM and propidium iodide (PI) were used to stain live (green) and dead (red) cells, respectively. Effective cell killing was observed when U87MG cells were exposed to ZnO_2_ NPs for 24 h (Figure 5C). The oxidative stress-triggered cancer cell apoptosis was then determined by annexin V-FITC/PI apoptosis detection kit. As shown in flow cytometry profiles (Figure 5D, Figure S4), U87MG cells underwent apoptosis after treatment with ZnO_2_ NPs for 12 h. Furthermore, the percentage of apoptotic cancer cells increased with increasing dose of ZnO_2_ NPs. The above results demonstrated the potential of pH-responsive ZnO_2_ NPs as an efficient therapeutic agent to cause oxidative stress and cancer cell apoptosis.

**Figure 6:**
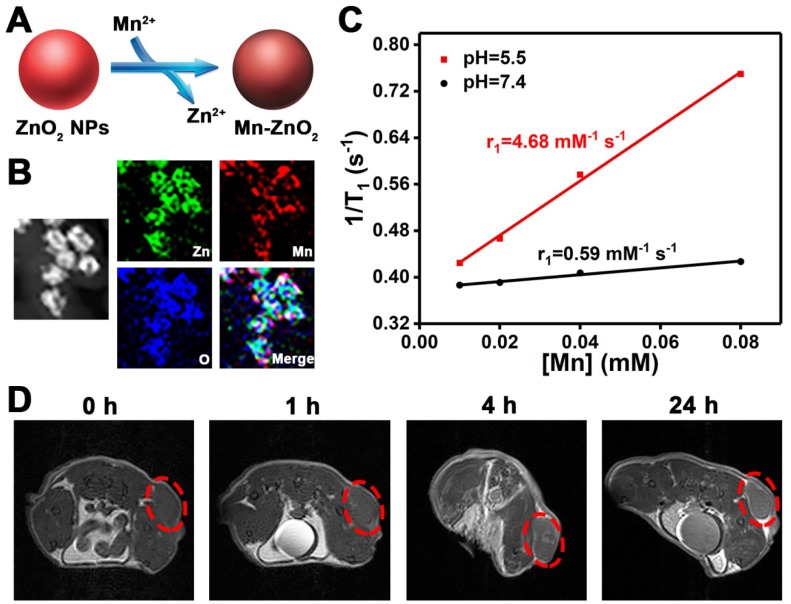
(A) Scheme showing the preparation of Mn-doped ZnO_2_ NPs through a facile cation-exchange approach. (B) EDS mapping of Mn-ZnO_2_ NPs. (C) The *r*_1_ values of Mn-ZnO_2_ NPs under different pH conditions. (D) *T*_1_-weighted MRI of U87MG tumor-bearing mice after intravenous injection of Mn-doped ZnO_2_ NPs. The red circles indicate the tumor area.

Theranostic agents that integrate diagnostic and therapeutic functions into a single nanoplatform offer great opportunities for personalized cancer treatment 53-59. In order to endow ZnO_2_ NPs with MRI contrast ability, a facile cation-exchange strategy was employed for the construction of Mn-doped ZnO_2_ NPs (Figure 6A). The multifunctional Mn-ZnO_2_ NPs were synthesized by mixing ZnO_2_ NPs with different amounts of MnCl_2_ at room temperature for 4 h. An obvious color change was observed after treatment of ZnO_2_ NPs with MnCl_2_ (Figure S5). Importantly, there was no distinct morphology difference between ZnO_2_ and Mn-ZnO_2_ NPs when the weight fraction of Mn was 6.5% or below (Figure S6). The Mn-ZnO_2_ NPs with 6.5% of Mn were thus selected for the following studies. Moreover, the energy-dispersive X-ray spectroscopy (EDS) and EDS mapping data also verified the successful fabrication of Mn-ZnO_2_ NPs (Figure 6B, Figure S7).

Inspired by the pH-dependent dissolution behavior of ZnO_2_ NPs, we compared the longitudinal (*T*_1_) relaxivity of Mn-ZnO_2_ NPs dispersed in buffer solutions with different pH values. Intriguingly, the *T*_1_ relaxation rate (*r*_1_) value increased from 0.59 to 4.68 mM^-1^ s^-1^ as the pH decreased from 7.4 to 5.5 (Figure 6C, Figure S8), revealing the pH-activated MRI contrast performance of Mn-ZnO_2_ NPs. Such an activatable *T*_1_ contrast effect is mainly due to the fact that pH-stimulated release of Mn^2+^ from Mn-ZnO_2_ NPs provides more efficient chemical exchange between Mn^2+^ ions and protons. The off-to-on contrast ability in response to specific stimuli makes Mn-ZnO_2_ NPs highly promising for tumor MRI *in vivo*. To verify this, U87MG tumor-bearing mice were injected with Mn-ZnO_2_ NPs through the tail vein and then imaged by an MRI scanner. As shown in Figure 6D, positive contrast effect was found at the tumor site after intravenous administration of Mn-ZnO_2_ NPs. Furthermore, the MRI *T*_1_ signal in the tumor region gradually increased with time, which could be attributed to the high tumor uptake of Mn-ZnO_2_ NPs. These results indicated that Mn-ZnO_2_ NPs is a promising MRI contrast agent for cancer imaging.

**Figure 7:**
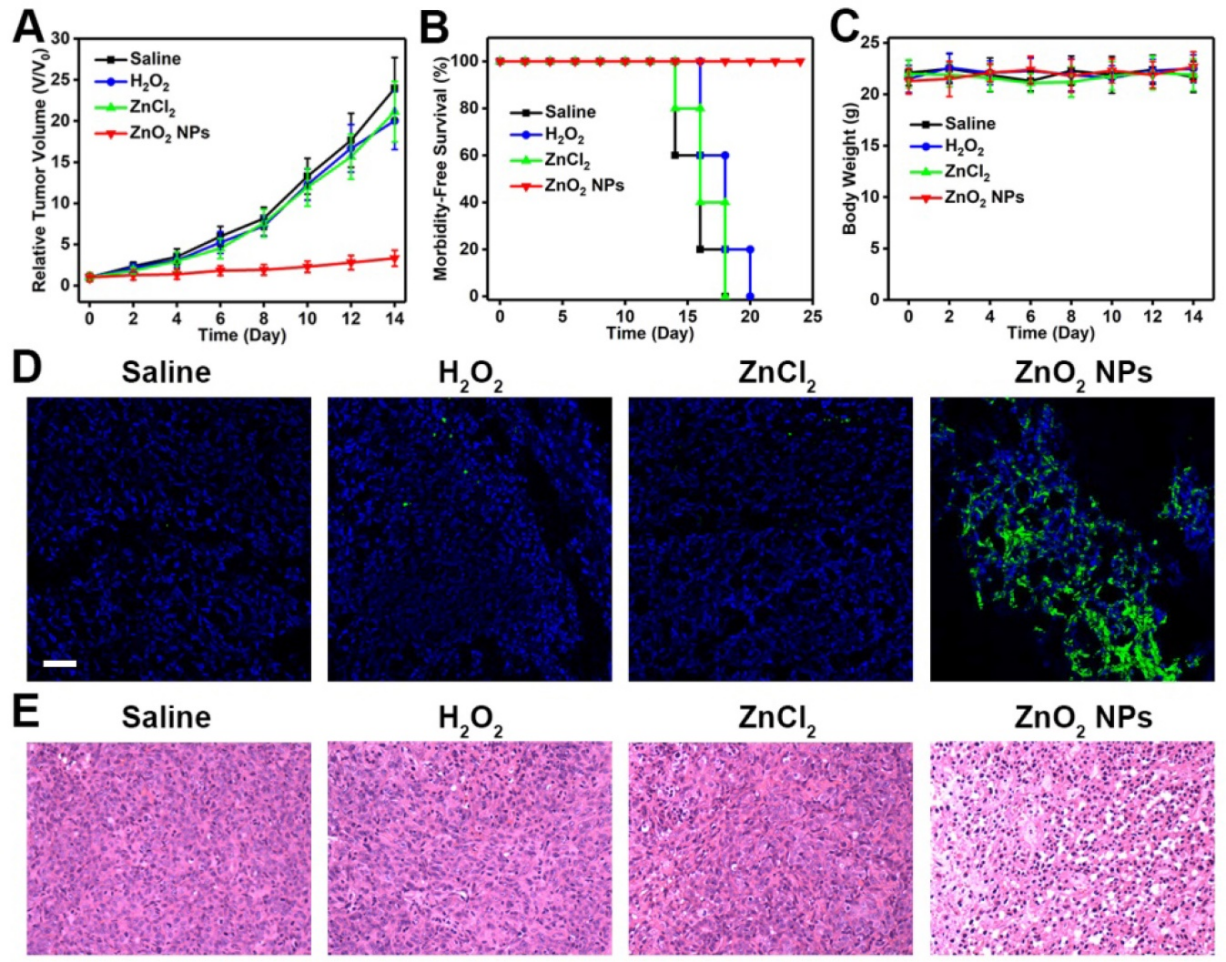
(A) Tumor growth curves of U87-bearing mice injected intravenously with different formulations. (B) Survival curves of mice in different groups. (C) Body weight changes of mice during the observation period. (D) TUNEL and (E) H&E staining of tumor tissues derived from different groups. Scale bar in d, 50 μm.

In view of the potent anticancer activity of PVP-modified ZnO_2_ NPs *in vitro*, we further investigated their feasibility for inhibiting tumor growth *in vivo*. Mice bearing U87MG tumors were treated with different formulations by intravenous injection, and tumor size was measured every other day using a caliper. As can be seen in Figure 7A, the growth of tumors in mice injected with ZnO_2_ NPs was significantly suppressed, demonstrating the high therapeutic efficacy of ZnO_2_-based oxidative stress inducers. In comparison, both H_2_O_2_ and ZnCl_2_ showed only limited tumor growth inhibition (Figure S9), most likely due to the rapid clearance after intravenous administration. Moreover, no significant body weight loss was observed after various treatments (Figure 7B,C). Hematoxylin and eosin (H&E)-stained images of major organs collected from different groups of mice at day 14 showed no obvious organ injury (Figure S10). Then, the oxidative stress-stimulated cancer cell apoptosis was determined by terminal deoxynucleotidyl transferase-mediated dUTP nick-end labeling (TUNEL) assay. Tumor tissues derived from mice treated with ZnO_2_ NPs had significantly more apoptotic cells as compared to that from saline-injected mice (Figure 7D). In addition, H&E staining indicated that tumors in ZnO_2_ NPs-treated mice exhibited severe damage (Figure 7E). The above results suggested that ZnO_2_ NPs are good candidates for cancer treatment *in vivo*.

## Conclusions

In summary, we have successfully developed a ZnO_2_ NPs-based ROS-generating nanoagent that enhances oxidative stress-mediated cancer cell killing through the collaboration of endogenous and exogenous ROS, and demonstrated its use in the fabrication of activatable theranostic nanoplatform for MRI-guided tumor treatment. After uptake by cancer cells, the pH-responsive ZnO_2_ NPs, in addition to releasing exogenous H_2_O_2_, also provide Zn^2+^ to facilitate the production of endogenous O_2_·^-^ and H_2_O_2_ from mitochondrial ETC, enabling highly effective synergistic tumor therapy. The pH-dependent dissociation behavior of ZnO_2_ NPs, together with the fact that cancer cells are more vulnerable to additional ROS than normal cells, allows them to efficiently trigger cell apoptosis in tumor and show negligible damage to major organs. Moreover, manganese could be easily doped into ZnO_2_ NPs by cation exchange to impart them with pH-activated MRI contrast property, which is appropriate for monitoring the dissolution of ZnO_2_ NPs and subsequent therapeutic process. Our study not only demonstrates the utilization of ZnO_2_ nanomaterials for theranostic applications, but also provides a two-pronged strategy to further improve oxidative stress-based cancer therapy by stimulating cooperation between endogenous and exogenous ROS.

## Supplementary Material

Supplementary figures and tables.

## Abbreviations

ROS: Reactive oxygen species
ZnO_2_ NPs: zinc peroxide nanoparticles
H_2_O_2_: hydrogen peroxide
O_2_·^-^: superoxide anion radical
ETC: electron transport chain
PVP: poly(vinylpyrrolidone)
MOPS: 3-(N-morpholino)propanesulfonic acid
DCFH-DA: 2',7'-dichlorofluorescin diacetate
MTT: thiazolyl blue tetrazolium bromide
PI: propidium iodide
Mn-ZnO_2_: Mn-doped ZnO_2_
MRI: magnetic resonance imaging
TEM: transmission electron microscopy
DLS: dynamic light scattering
XPS: X-ray photoelectron spectroscopy
DCFH-DA: 2',7'-dichlorofluorescin diacetate
H&E: hematoxylin and eosin
TUNEL: terminal deoxynucleotidyl transferase-mediated dUTP nick-end labeling
