# Supplementary material for: Cooperation of endogenous and exogenous reactive oxygen species induced by zinc peroxide nanoparticles to enhance oxidative stress-based cancer therapy
Source: Theranostics. 2019 Sep 23;9(24):7200–9. doi: 10.7150/thno.39831 (PMC6831298; doi:10.7150/thno.39831)
Supplement: Supplementary file 1 — Supplementary figures and tables. [file thnov09p7200s1.pdf]

Supporting Information

Cooperation of endogenous and exogenous reactive oxygen species induced by zinc peroxide nanoparticles to enhance oxidative stress-based cancer therapy

Li-Sen Lin[1,2*], Jun-Feng Wang[3*], Jibin Song[4*], Yijing Liu[2], Guizhi Zhu[2], Yunlu Dai[2], Zheyu Shen[2], Rui Tian[2], Justin Song[2], Zhantong Wang[2], Wei Tang[2], Guocan Yu[2], Zijian Zhou[2], Zhen Yang[2], Tao Huang[5], Gang Niu[2], Huang-Hao Yang[4], Zhi-Yi Chen[1]✉, Xiaoyuan Chen[2]✉

[1]Department of Ultrasound Medicine, Laboratory of Ultrasound Molecular Imaging, the Third Affiliated Hospital of Guangzhou Medical University, Guangzhou 510150, China

[2]Laboratory of Molecular Imaging and Nanomedicine (LOMIN), National Institute of Biomedical Imaging and Bioengineering (NIBIB), National Institutes of Health (NIH), Bethesda, Maryland 20892, United States

[3]Department of Ultrasound, the First Affiliated Hospital of Harbin Medical University, Harbin, Heilongjiang 150076, China

[4]MOE Key Laboratory for Analytical Science of Food Safety and Biology, College of Chemistry, Fuzhou University, Fuzhou 350108, China

[5]Department of Radiology, the Fourth Hospital of Harbin Medical University, Harbin, Heilongjiang 150076, China

[*]These authors contributed equally to this work.

✉Corresponding authors: Zhi-Yi Chen (winchen@vip.126.com), Xiaoyuan Chen (shawn.chen@nih.gov)

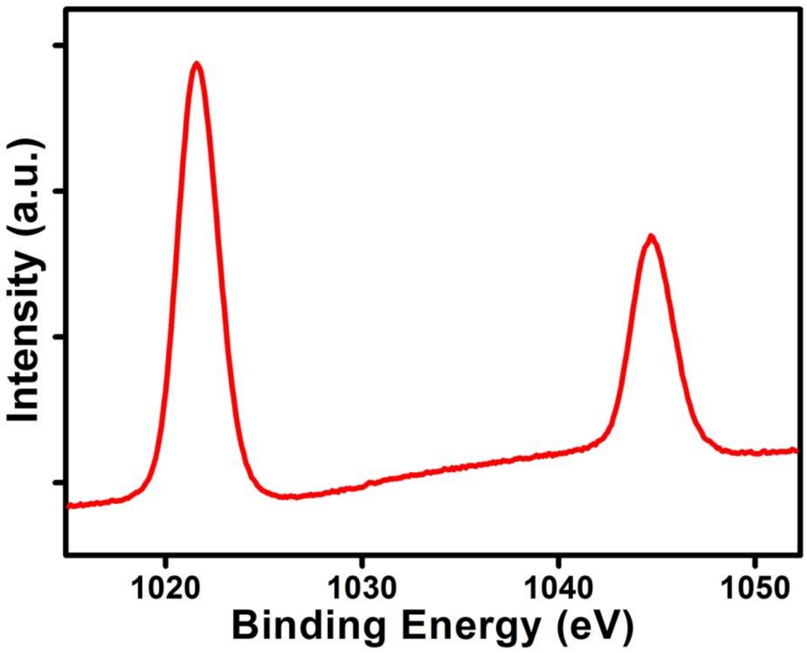

Figure S1. Zn 2p X-ray photoelectron spectroscopy (XPS) spectra of ZnO_2 NPs.

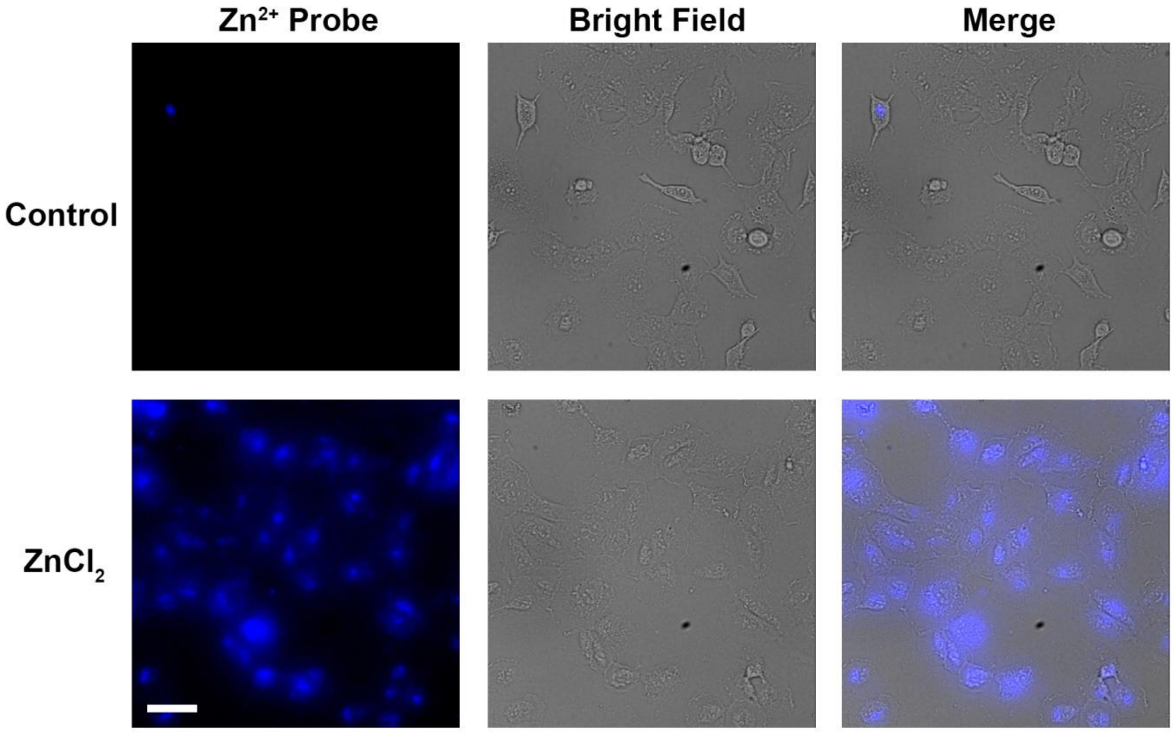

Figure S2. Fluorescence images of zinquin ethyl ester-stained U87MG cells after incubation with 100 μM $ZnCl_2$ for 4 h. Scale bar, 50 μm.

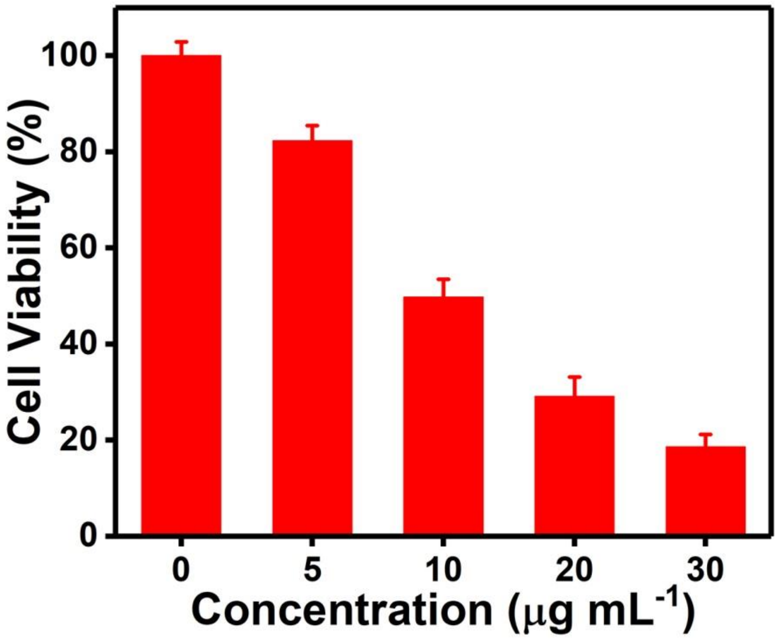

Figure S3. *In vitro* anticancer activity of ZnO_2 NPs after 48 h of incubation.

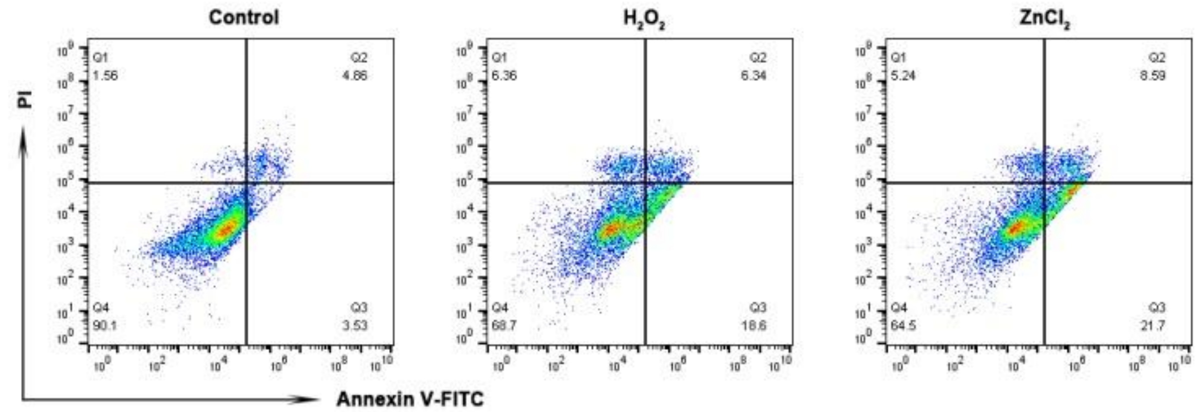

Figure S4. Flow cytometry data showing apoptosis in U87MG cells after incubation with 200 μM H_2O_2 or 200 μM $ZnCl_2$ for 12 h.

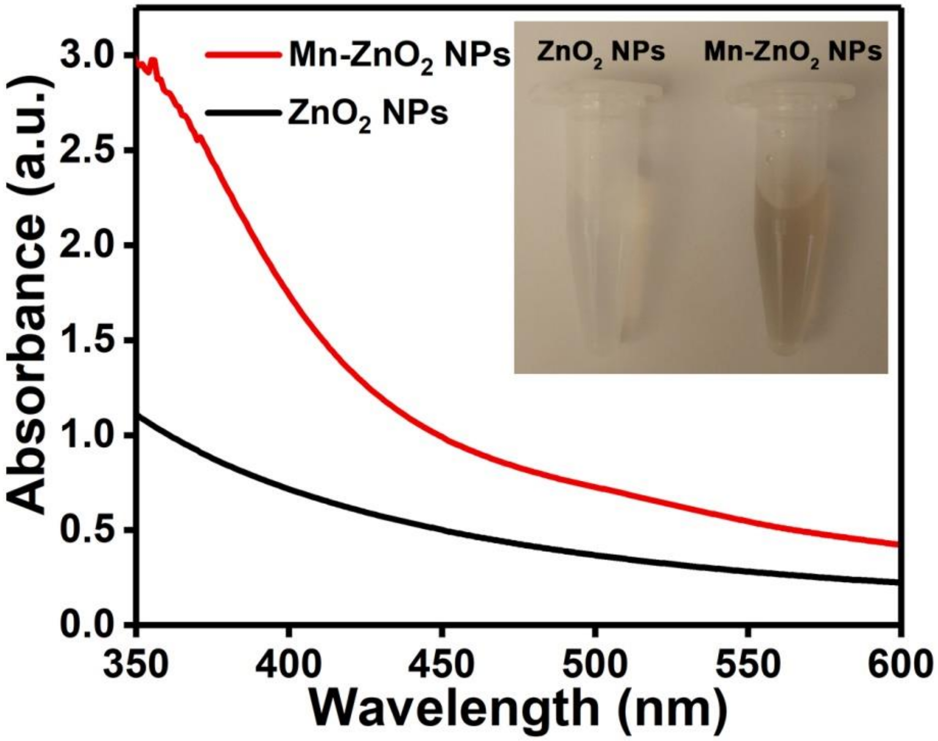

Figure S5. UV/Vis absorption spectra of ZnO_2 NPs before and after Mn-doping.

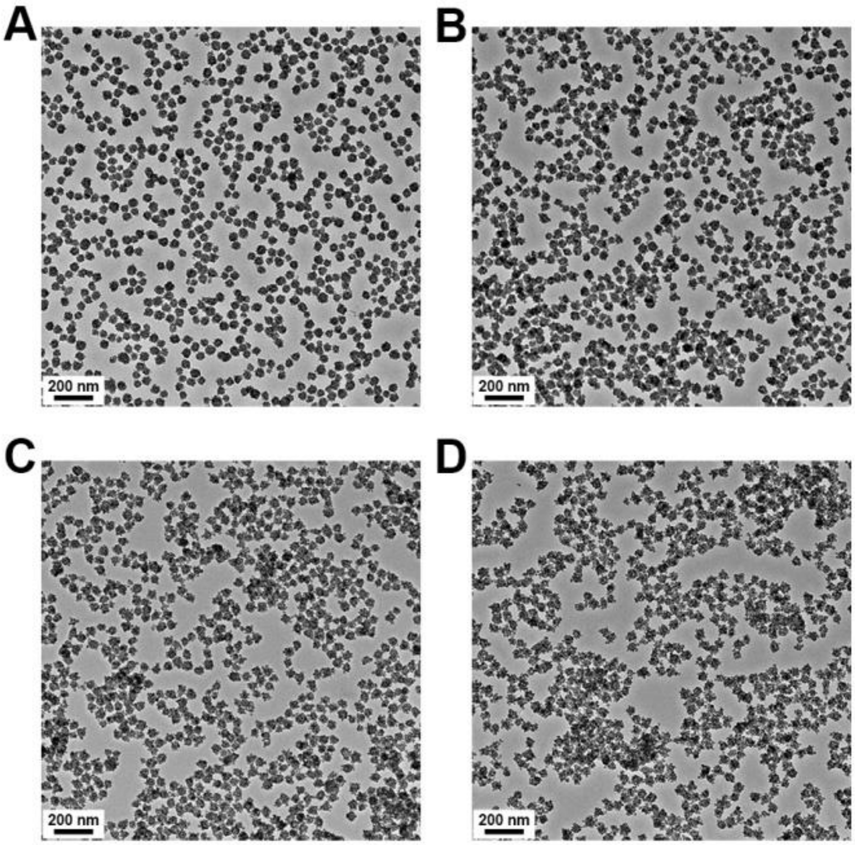

Figure S6. TEM images of Mn-ZnO_2 NPs with different ratio of Mn. The weight fraction of Mn: (A) 0%, (B) 4.3%, (C) 6.5%, and (D) 10.6%.

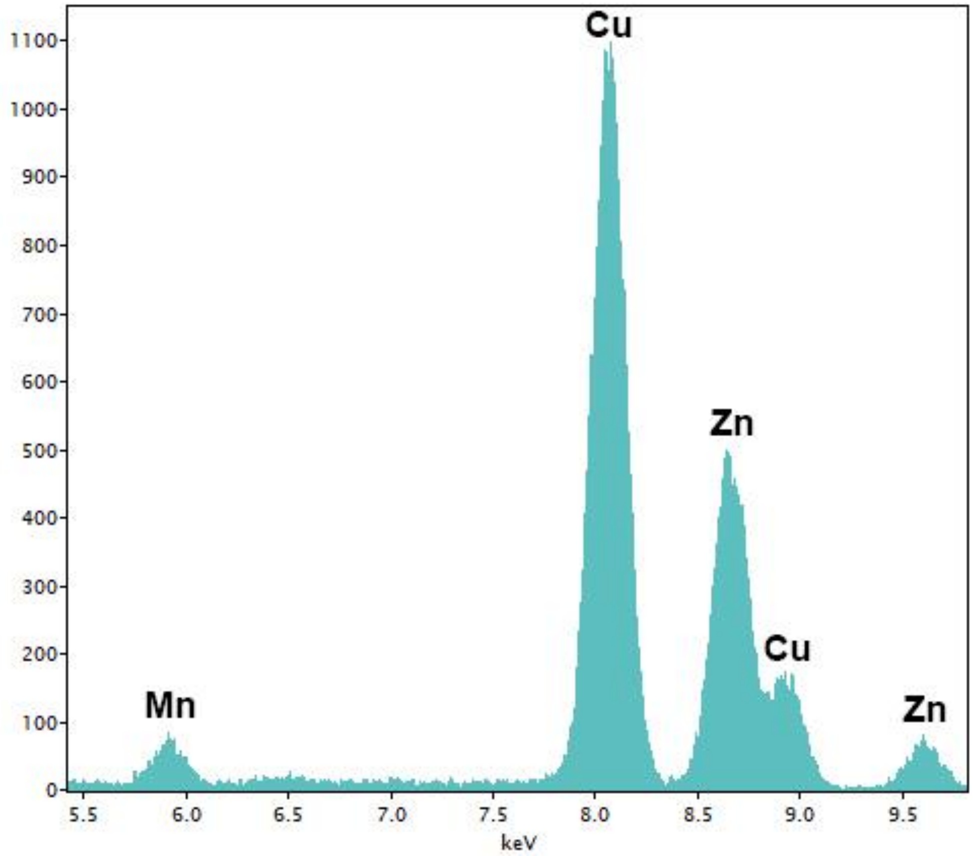

Figure S7. EDS spectrum of Mn-ZnO_2 NPs. Note that the signal of Cu is from the copper grid.

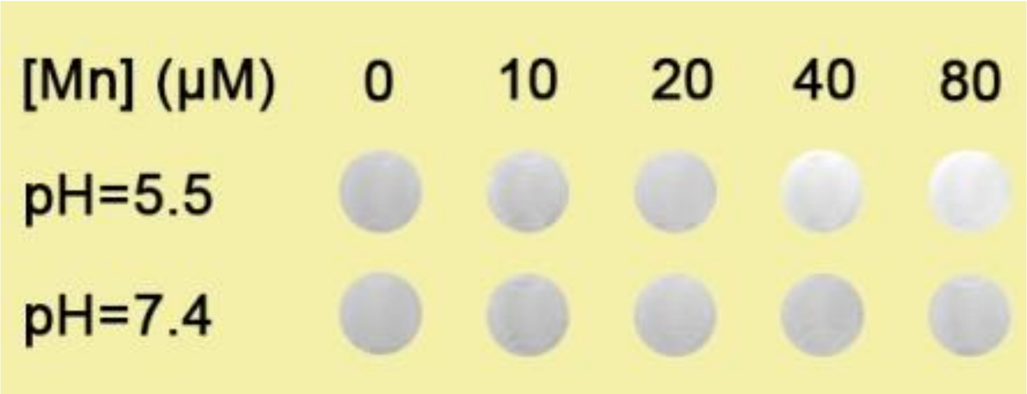

Figure S8. T_1-weighted MRI images of Mn-ZnO_2 NPs under different pH conditions.

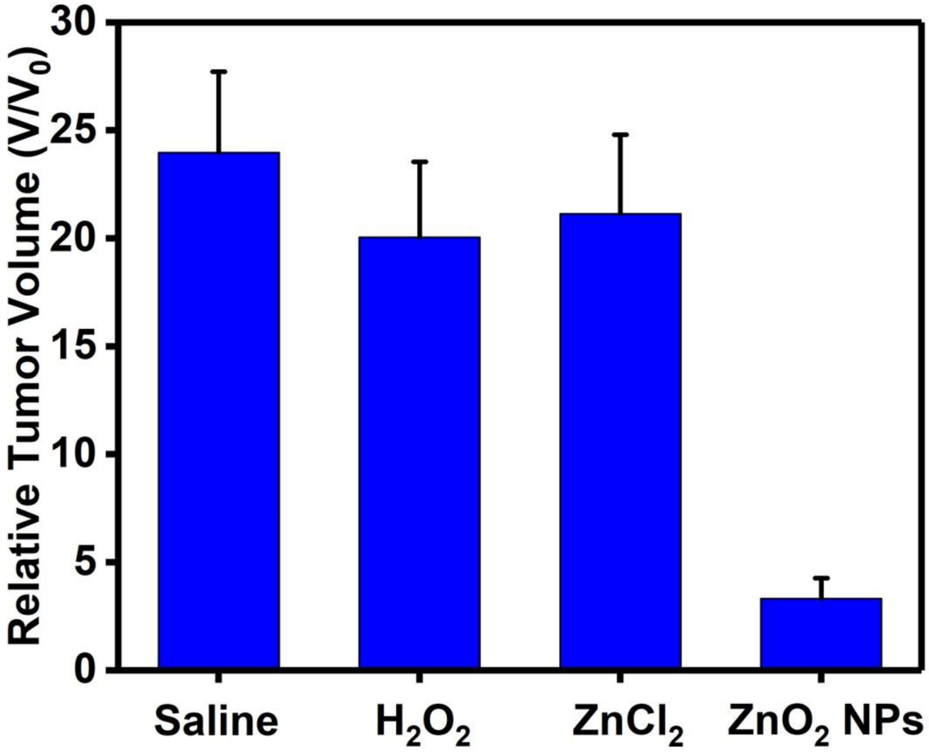

Figure S9. Tumor sizes at day 14 in different groups.

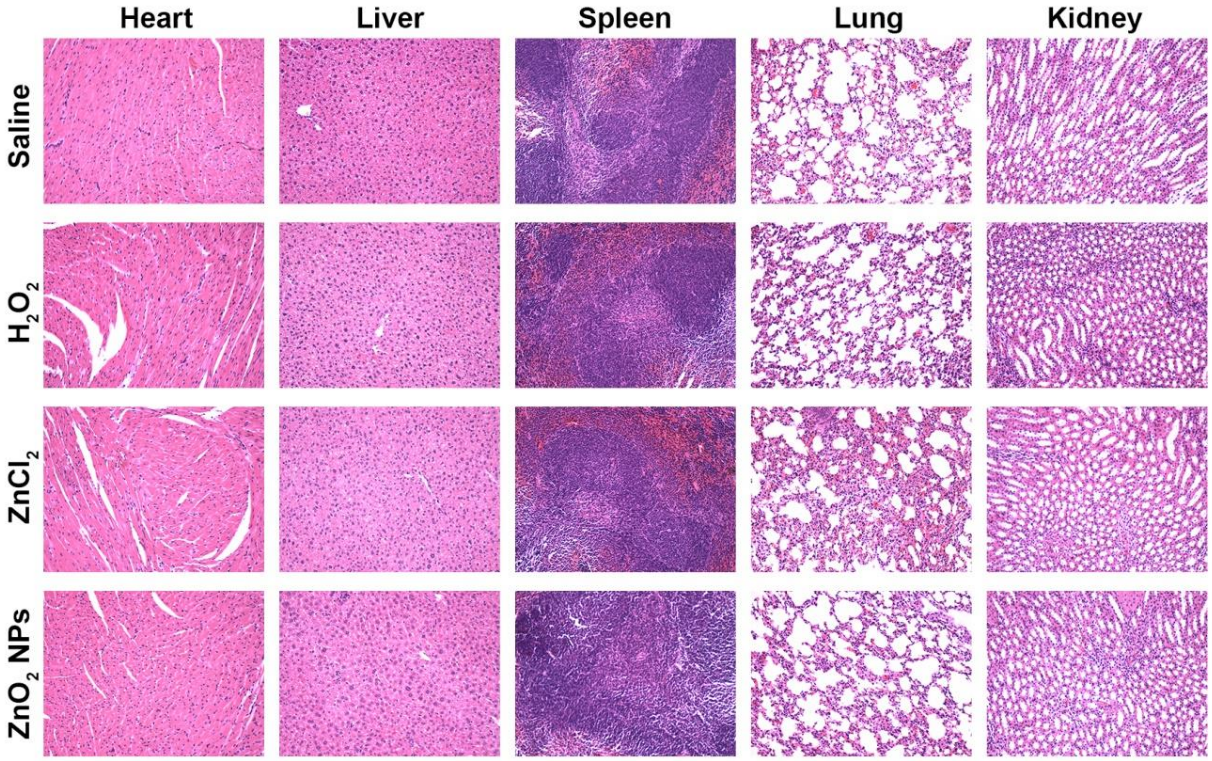

Figure S10. H&E-stained images of major organs collected from different groups of mice at day 14.
